# How to Improve Behavioral Parent and Teacher Training for Children with ADHD: Integrating Empirical Research on Learning and Motivation into Treatment

**DOI:** 10.1007/s10567-020-00327-z

**Published:** 2020-09-24

**Authors:** Saskia van der Oord, Gail Tripp

**Affiliations:** 1 Behavior, Health and Psychopathology, KU Leuven, Tiensestraat 102, 3000 Leuven, Belgium; 2 Developmental Psychology, University of Amsterdam, Nieuwe Achtergracht 129, 1018 WS Amsterdam, The Netherlands; 3 Human Developmental Neurobiology Unit, Okinawa Institute of Science and Technology Graduate University, 1919-1 Tancha, Onna, Kunigami District, Okinawa Prefecture 904-0495 Japan

**Keywords:** Attention deficit hyperactivity disorder, Learning, Reward, Treatment, Behavioral parent training

## Abstract

Attention deficit hyperactivity disorder [ADHD] is one of the most common psychiatric disorders of childhood with poor prognosis if not treated effectively. Recommended psychosocial evidence-based treatment for preschool and school-aged children is behavioral parent and teacher training [BPT]. The core elements of BPT are instrumental learning principles, i.e., reinforcement of adaptive and the ignoring or punishment of non-adaptive behaviors together with stimulus control techniques. BPT is moderately effective in reducing oppositional behavior and improving parenting practices; however, it does not reduce blinded ratings of ADHD symptoms. Also after training effects dissipate. This practitioner review proposes steps that can be taken to improve BPT outcomes for ADHD, based on purported causal processes underlying ADHD. The focus is on altered motivational processes (reward and punishment sensitivity), as they closely link to the instrumental processes used in BPT. Following a critical analysis of current behavioral treatments for ADHD, we selectively review motivational reinforcement-based theories of ADHD, including the empirical evidence for the behavioral predictions arising from these theories. This includes consideration of children’s emotional reactions to expected and unexpected outcomes. Next we translate this evidence into potential ADHD-specific adjustments designed to enhance the immediate and long-term effectiveness of BPT programs in addressing the needs of children with ADHD. This includes the use of remediation strategies for proposed deficits in learning not commonly used in BPT programs and cautions regarding the use of punishment. Finally, we address how these recommendations can be effectively transferred to clinical practice.

## Introduction

Attention deficit/hyperactivity disorder [ADHD] (American Psychiatric Association 2013) is one of the most common psychiatric disorders of childhood with poor prognosis if not treated effectively (Tarver et al. 2014). Evidence-based treatments for ADHD include pharmacological and non-pharmacological therapies (Evans et al. 2014). Pharmacological interventions have well-established efficacy for ADHD symptom reduction, but not necessary for associated difficulties. Moreover long-term effects may be limited, side-effects experienced, and parents often have a preference for non-pharmacological options (Daley et al. 2014; Johnston et al. 2008; Lee et al. 2012; Van der Oord et al. 2008).

The primary evidence-based, and most common, non-pharmacological treatment for preschool and elementary school-aged children with ADHD is Behavioral Parent and Teacher Training [BPT] (Daley et al. 2018; Evans et al. 2018). However, a recent meta-analysis showed that although BPT is effective in reducing oppositional behaviors, improves positive parenting, reduces negative harsh parenting and increases parent’s feelings of competence regarding their parenting, it does not significantly reduce independent ratings of ADHD symptoms (Daley et al. 2014). Furthermore, effect sizes for the reduction of behavioral problems and improved parenting are modest and generally dimish after discontinuation (Lee et al. 2012). Additionally, an extensive meta-analysis of the effects of psychosocial treatments over time (including mostly behavioral treatments) shows that in the last 50 years effect sizes for treatments of ADHD have not improved, but rather show a non-significant decline in effectiveness (Weisz et al. 2018). All in all, this calls for exploring how our current BPTs for ADHD may be improved. One potential way is the integration of research on causal processes in ADHD into behavioral treatment.

Core elements of BPT are instrumental learning principles. Parents and teachers are taught, as much as possible, to continuously reinforce adaptive and punish or ignore non-adaptive behavior (consequent techniques) and to increase the discriminative value of the stimuli predicting adaptive behavior (antecedent or stimulus control techniques) (Van der Oord and Daley 2015). Most of the available behavioral parent training programs were developed in the 1970s and 1980s (e.g., Helping the non-compliant child, (Forehand and McMahon 1981); Parenting your defiant child (Kazdin 2005); The Incredible Years parent training, (1992); Defiant Children, (Barkley 1987); Triple-P, (Sanders 1992; Sanders and Dadds 1993)) for children with behavioral problems in general (Shaffer et al. 2001), not specifically for children with ADHD. Multiple RCTs were conducted to test the effectiveness of these parenting programs with children with disruptive behavior disorders. Results were generally positive, with typically moderate to large effects sizes for the reduction of disruptive behavior, establishing behavioral parenting training as an effective treatment of children with disruptive behavior disorders (Kaminski and Claussen 2017; Leijten et al. 2019). Historically, ADHD was classified as a disruptive behavior disorder together with oppositional defiant disorder (ODD) and conduct disorder (CD). However, with the publication of DSM-5 ADHD, but not ODD and CD, is included in the new category of Neurodevelopmental Disorders, reflecting a life-span perspective and increased understanding of the neural correlates of the disorder (Doernberg and Hollander 2016).

Originally developed for children with disruptive behavior disorders, most of these parenting programs do not address these specific causal processes in ADHD in their use of instrumental and stimulus control techniques. Insight into underlying deficits and causes of ADHD can inform which components of our behavioral treatments may be more effective or how these elements can be targeted for the needs of children with ADHD (Antshel and Barkley 2008; Chacko et al. 2014; Emmelkamp et al. 2013; Sonuga-Barke and Halperin 2010; Weisz et al. 2018).

Purported underlying causal mechanisms in ADHD are heterogeneous, with, among others, deficits in motivational processes (e.g., reward and punishment sensitivity), cognition, timing, and emotional lability proposed as distinct but partly overlapping pathways toward the behaviors that define ADHD (Dovis et al. 2012; Sjöwall et al. 2013; Sonuga-Barke et al. 2010). The proposed deficits in reinforcement processes in ADHD may be especially relevant to improving the instrumental techniques used in BPT; they link closely to the core principles of behavior management (reward and punishment) and there is ample neurobiological and behavioral evidence for altered motivation (especially reward sensitivity) in ADHD (Fosco et al. 2015; Luman et al. 2005, 2010).

**Fig. 1:**
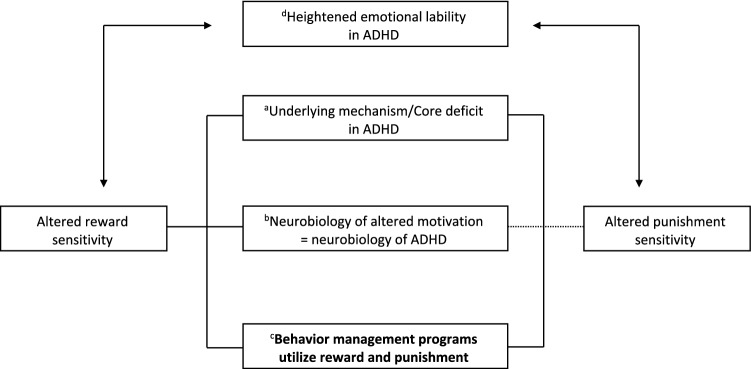
A framework for understanding the importance of altered motivational processing to the neurobiology of ADHD and its psychosocial management: (a) Altered sensitivity to reward is considered by many to be a core deficit in ADHD, potentially underlying the symptoms of the disorder itself, evidence for altered sensitivity to punishment is limited; (b) The neural circuitry of reinforcement and reinforcement learning is well established—offering insight into the neurobiology of ADHD itself. Research with typically developing individuals provides some insight into brain regions relevant to the processing of punishment; (c) Reward and punishment (at least response cost) are the essence of behavior management programs. Differences in their processing in ADHD should be considered in BPT for this group; (d) Increased emotional lability in ADHD likely contributes to and is influenced by altered sensitivity to consequences

Figure 1 highlights the importance of altered motivational processing to both understanding the neurobiology of ADHD and improving its behavioral management. Evidence is accumulating that children with ADHD differ from Typically Developing [TD] children in their response to motivationally significant events, i.e., reward and punishment. This has led researchers to question if altered reinforcement processing is a core deficit underlying symptoms of ADHD. Given the complexity of the disorder it is unlikely that there is one single pathway to ADHD. However, the neurobiology of reinforcement and reinforcement learning is well documented and may offer important clues regarding the neurobiological pathways to ADHD (Tripp and Wickens 2009). The neurobiology of punishment is currently less well mapped (Jean-Richard-Dit-Bressel et al. 2018), especially in ADHD. Behavioral treatment programs for ADHD rely heavily on the use of reinforcement to shape and increase rates of appropriate behavior together with mild punishment to deter unwanted behaviors. Improving the efficacy of behavior management necessitates reviewing the use of reward and punishment in behavioral management of children with ADHD based on research findings. In addition, evidence of emotional lability and emotional dysregulation in ADHD (Graziano and Garcia 2016; Sobanski et al. 2010) may be an important process to consider in behavioral treatment for ADHD. Such emotional responsiveness may be intertwined with responses to reward and punishment (Amsel 1958; Brotman et al. 2017), especially in ADHD; i.e., exacerbated in response to punishment or the failure to receive anticipated rewards (i.e., Amsel’s frustration theory, (Amsel 1958, 1962, 1992b)).

Furthermore, experimental research on extinction in ADHD (what happens when reward is removed once behavior is learned) is potentially relevant not only for how non-adaptive behavior can be extinguished (e.g., ignoring mild misbehavior, with the purpose of reducing its frequency) but also potentially for how to improve long-term effectiveness of behavioral treatment for ADHD. Behavioral treatment for ADHD is largely based on teaching adaptive behavior through, as much as possible, continuously rewarding such behavior; however, in daily life such high rates of reinforcement cannot be maintained. Some behavioral treatment programs explicitly include fading procedures following initial high frequencies of reinforcement, others don’t (Hornstra et al. 2020). Thus, once the behavioral treatment ends the target behavior may no longer be rewarded, or rates of reinforcement may decline, although the general idea is that the learned behavior should persist after the treatment (behavioral persistence in extinction). Thus, extinction processes in ADHD can potentially also be informative for promoting the long-term effectiveness of behavioral treatment for ADHD.

Behavior therapy is defined as being *based upon* empirical psychology and *striving toward* continuous development (Margraf 1998). The body of empirical evidence for specific deficits in motivation or altered processing of reinforcement (Luman et al. 2005, 2010; Smith and Langberg 2018) has not, to our knowledge, led to adaptation of the core elements or delivery of BPTs in ADHD. In other areas of psychopathology evidence from experimental studies on core deficits and treatment methods has clearly changed the way the basic elements of behavioral treatments are implemented (Emmelkamp et al. 2013; Wittchen et al. 2014). For example, understanding of how to conduct exposure for anxiety disorders has changed dramatically based on empirical evidence of prediction error/inhibitory learning (Craske et al. 2014). Potentially as a result, effect sizes for the treatment of anxiety in children have improved over the last 50 years (Weisz et al. 2018).

This is not to say there have been no important developments in the way BPT is provided for children with ADHD in the last decades. Some have addressed parents, or subgroups of parents, ability to access and use the principles of BPT, targeting enhanced uptake, attendance and adherence (Chacko and Scavenius 2018; Chacko et al. 2012; Chronis et al. 2004), others have focused on methods of delivering the techniques in BPT (e.g., through videotape modeling (Webster-Stratton 1992) or remote delivery (Vander Stoep et al. 2018)) and efforts have been made to adapt BPT for different cultural groups (Thompson et al. 2017). Other examples include the development of a version of BPT that also targets emotional and depressive problems in the parents of children with ADHD (Chronis-Tuscano et al. 2013); versions of BPT specifically for fathers of children with ADHD (Fabiano et al. 2012), or for parents who have ADHD themselves (Jans et al. 2015). However, to our knowledge the core instrumental procedures promoted in the BPT manuals have not changed. The one parenting program that developed a different approach toward changing the behavior of the child is the New Forest Parenting Program (NFPP) for preschoolers with ADHD (Thompson et al. 2009). This program, developed specifically for parents of children with ADHD, targets deficits in the child’s self-regulation and the parent–child relationship (e.g., teaching parents to scaffold self-regulation through joint play), but other than teaching the child to deal with delay (based on the delay aversion theory of ADHD see later (Sonuga-Barke 2003)), it does not integrate findings of other motivational deficits in ADHD into the behavioral management techniques taught, e.g., the way parents are taught to apply reward or punishment.

There clearly seems to be a research-practice gap given that a sizable body of empirical and theoretical work has been conducted on motivational deficits in ADHD (for reviews see Luman et al. 2005, 2010; Smith and Langberg 2018; note there is much less research available on punishment). Therefore in the current paper, we first discuss the core elements of most behavioral parent/teacher training programs used in samples of children with ADHD, next we highlight the relevant motivational accounts of ADHD for BPT (i.e., those that focus on instrumental learning in ADHD) their predictions for the behavior of children with ADHD in response to reward and punishment, and the empirical evidence for the behavioral predictions arising from these theories. Finally, we translate this evidence into potential ADHD-specific adjustments for BPT programs.

## Core Elements of Behavioral Parent and Teacher Training (for ADHD)

The foundation for any treatment is psycho-education about the specific disorder, in this case ADHD, and for BPT, information on (social) learning theory and behavior management principles (see next paragraph). The primary aim of psycho-education for ADHD is to increase parents’ knowledge about the nature of ADHD, it’s possible causes, and the potential treatment options (Daley et al. 2018). As most of the BPT programs were not developed for children with ADHD some programs have moved to introduce ADHD-specific psycho-education (e.g., Forehand and Long 2002). We have not systematically reviewed the content of such psycho-education as it is often not published (manuals typically target behavior disorders generally). However, the information provided on underlying causal mechanisms generally seems more focused on deficits in executive functioning and working memory. There is less attention to altered sensitivity to reward, and even less about responsiveness to punishment (see, e.g., reviews of Antshel and Barkley 2008; Chacko et al. 2014), although behavioral descriptions of the responses of children with ADHD to instrumental techniques often reference such altered responding.

Next to psycho-education, generic operant/instrumental learning principles (Skinner 1938) are core elements of every BPT training program. Other important influences include Bandura’s work on social learning, e.g., the importance of modeling in learning and problem solving (Bandura 1971). Instrumental learning is the learning of the contingency between behavior and outcome in a certain situation. The core idea is that the behavior of an organism is shaped by a discriminative stimulus (antecedent or stimulus that comes before the behavior) and the consequences of the behavior. Behavior change can be bought about by either changing the discriminative value of the discriminative stimulus (e.g., by reducing other competing stimuli in the environment or increasing its saliency; stimulus control) or by changing the consequences of the behavior. Behavioral management programs such as a Daily Report Card or a token economy system are a combination of both stimulus control and consequent techniques. Stimulus control techniques used in BPT include for example giving clear instructions, providing clear rules and more structure (in time and space). The consequences of the behavior can either decrease or increase the frequency of its occurrence; reinforcement increases the frequency of a behavior, punishment decreases the frequency of a behavior. Both can be either positive (introducing something) or negative (omitting/taking something away—the expected consequence stays away).

Alongside such instrumental learning, Pavlovian learning (classical conditioning; the learning of associations) is intertwined with the expression of problem behavior and it’s management by behavioral treatment. In classical conditioning an originally neutral stimulus becomes a predictor of another event, sometimes developing an emotional connotation. For example, the instruction of a teacher to start math can, due to previous negative reactions of the teacher to the child during the math class, become a conditioned stimulus that triggers feelings of worthlessness (a conditioned emotional response) and subsequent behavioral problems (e.g., clowning; a conditioned behavioral/avoidance response) in an attempt to escape such feelings/situations. This clowning can then be reinforced by its consequences (e.g., laughter of children in the class; positive reinforcement). In designing specific (often instrumental) interventions for a child, these Pavlovian learning processes are important. At the same time parents and teachers implementing behavioral treatment have their own learning histories that shape their willingness to use techniques recommended by behavior therapists. Without addressing their attitudes toward specific techniques a parent or a teacher will not be able to convincingly implement instrumental interventions for a child. Thus the basis for effective behavior therapy is comprehensively assessing or addressing the instrumental and classical components of the child’s problem behavior and the learning history of parents/teachers. Individual case conceptualizations and functional analysis take into account these processes and are the basis for effective BPT training(Chronis et al. 2004). Table 1 (Hornstra et al. 2019) provides an overview of the variety of techniques that can be used in BPT for ADHD (this taxonomy was developed on the basis of leading reviews Chorpita and Daleiden 2009; Kaminski et al. 2008; Lee et al. 2014; Michie et al. 2013)). Not all behavioral parent or teacher training programs use all of these elements or use these elements with the same frequency. Some focus more on stimulus control techniques or antecedent techniques, others focus more on contingency management (Hornstra et al. 2020; Leijten et al. 2019). A recent meta-analysis on effective elements of BPT showed that in samples of children with behavioral disorders (not specific ADHD samples) the inclusion of positive reinforcement, praise, time-out and natural consequences was associated with enhanced effectiveness of these treatment programs (Leijten et al. 2019).^1^ However, as children with ADHD are suggested to have specific deficits in learning from reward and punishment (see the paragraph below for the motivational accounts of ADHD), it is still to be determined if similar elements in BPT have equal enhancing effects in samples of children with ADHD. Our paper may, based on evidence from experimental studies, provide guidance for whether certain elements of BPT may be more or less appropriate for children with ADHD.

**Table 1:** Taxonomy of techniques and components of behavioral parent and teacher training programs for children with ADHD (Hornstra et al. 2019)

Number	Name	Definition
1. Shaping knowledge		
1.a	Psycho-education parent	The formal review of information with the caretaker(s) about the development of the child’s problem and its relation to a proposed intervention. This often involves an emphasis on the caretaker’s role in either or both
1.b	Psycho-education teacher	The formal (usually didactic) review of information, directed toward the child’s teacher(s), or school
2. Observation and monitoring		
2.a	Monitoring	Establish a method for the person to monitor and record their own behavior(s) or the behavior(s) of the child
2.b	Behavioral/functional analysis	Explain, teach or train parents/teacher how to identify and test hypotheses about the behavior, its causes and consequences, by collecting and interpreting data
3. Antecedents		
3.a	Disciplinary communication	Setting limits and rules and/or giving clear and developmentally appropriate directions, stating behavioral expectations and consequences
3.b	Anticipate and plan for misbehavior	Thinking ahead about problem situations and prepare a plan of behavior management for the child before entering the potential problem situation
3.c	Restructuring the environment	Change, or advise to change the physical or social environment in order to facilitate performance of the wanted behavior, create barriers to the unwanted behavior (other than prompts/ cues, rewards or punishments) or avoid exposure to specific social, contextual/physical cues for the behavior, including changing daily or weekly routines
3.d	Prompt/cues	Introduce or define environmental or social stimulus with the purpose of prompting or cueing the behavior. The prompt or cue would normally occur at the time or place of performance. Includes also the removal of prompts or cues (fading)
3.e	Distraction	Advise or arrange to use an alternative focus for attention to avoid triggers for unwanted behavior
3.f	Behavior substitution	Prompt substitution of the unwanted behavior with a wanted or neutral behavior
3.g	Habit formation	Prompt rehearsal and repetition of the behavior in the same context
4. Consequences/contingency management		
Positive consequences		
4.a	Social reward	The training of parents or others involved in the social ecology of the child in the administration of social rewards to promote desired behaviors. This can involve praise, encouragement, affection, or physical proximity
4.b	Material reward (behavior)	Arrange for the delivery of money, vouchers or other valued objects if and only if there has been effort and/or progress in performing the behavior (includes ‘Positive reinforcement’)
4.c	Activity reward	Arrange for the delivery of a preferred activity if and only if there has been effort and/or progress in performing the behavior
4.d	Reward (not specified)	Arrange delivery of a reward if and only if there has been effort and/or progress in performing the behavior (not specified as social/material/activity)
4.e	Remove aversive stimulus/ punishment	Advise or arrange for the removal of an aversive stimulus or an unpleasant consequence to facilitate behavior change (includes Escape learning, negative reinforcement)
4.f	Reward approximation	Arrange for reward following any approximation to the target behavior gradually rewarding only performance closer to the want behavior (includes Shaping)
4.g	Situation-specific reward	Arrange reward following the behavior in one situation but not in another (includes Discrimination training)
4.h	Reward alternative/incompatible behavior	Arrange reward for responding in a manner that is incompatible with a previous response to that situation (includes Counter-conditioning) or for performance of an alternative to the unwanted behavior (includes differential reinforcement)
4.i	Reduce reward frequency/stretching the ratio’s	Arrange reward to be made contingent on increasing duration or frequency of the behavior (includes Thinning) gradually weaning the density of reinforcement during acquisition from very high levels (100%) to very spare levels of reward (e.g., 20%)
4.j	Consistent responding	Teach parents the importance of consistent responses to child behavior
5. Consequences/ contingency management		
Negative consequences		
5.a	Planned ignoring	Parents or teachers are instructed to ignore frequently occurring, mildly annoying behaviors that serve the function of attention seeking
5.b	Natural and logical consequences	Training for parents or teachers in (a) allowing youth to experience the negative consequences of poor decisions or unwanted behaviors (e.g., getting cold for not wearing a hat), or (b) delivering consequences in a manner that is of appropriate level and type for the behavior performed by the child
5.c	Punishment	Arrange for an aversive consequence contingent on the performance of the unwanted behavior other than natural and logical consequences, response cost or (over)correction
5.d	Response cost	Remove or discontinue reinforcement following performance of an undesirable or disruptive behavior
5.e	(Over)correction	Parents or teacher are instructed to designate the unwanted behavior or repeat the wanted behavior in an exaggerated way following an unwanted behavior
6. Combined techniques		
6.a	Daily Report Card [DRC]	The use of a list of behaviors by the teacher that have been deemed appropriate targets for intervention (e.g., interrupting, non-compliance, academic productivity, academic engagement). Associated with each item is a means of rating the target behavior across one or more observation intervals (e.g., time of day or class period). DRC forms are sent home with the child each day, and parents review daily and weekly progress and provide home-based privileges (e.g., use of bicycle, computer time) contingent on meeting goals
6.b	Tangible rewards/token economy	The training of parents or others involved in the social ecology of the child in the administration of tangible rewards to promote desired behaviors. This can involve a point or token system
6.c	Time-out	The training of or the direct use of a technique involving removing the youth from all reinforcement for a specified period of time following the performance of an identified, unwanted behavior
6.d	Premack principle	A more-preferred activity can be used to reinforce a less-preferred activity
7. Generalization and maintenance		
7.a	Rehearsal, role-playing, practice or visualize (parent/teacher)	In-session opportunities for parents to practice skills through rehearsal and role-playing situations: role-playing with the parent trainer
7.b	Homework	A set of tasks assigned by the therapist to do at home
7.c	Modeling	Provide an observable sample of the performance of the behavior, directly in person or indirectly (e.g., via film, pictures, for the person to aspire or to imitate), includes modeling
7.d	Generalization of target behavior (parent/teacher)	Advise to perform the wanted behavior, which is already performed in a particular situation, in another situation
7.e	Maintenance/relapse prevention	Exercises and training designed to consolidate skills already developed and to anticipate future challenges that might arise after termination or reduction of services
7.f	Problem solving (parent/teacher)/Conjoint behavioral consultation	Techniques, discussions, or activities designed to bring about solutions to targeted problems, usually with the intention of imparting a skill for how to approach and solve future problems in a similar manner, using following steps: Identifying the Problem, Defining the Problem, Forming a Strategy, Organizing Information, Allocating Resources, Monitoring Progress, Evaluating the Results
8. Relationship building communication skills		
8.a	Emotional communication	Using relationship-building communication skills (e.g., active listening); helping children identify and appropriately express emotions
8.b	Positive interactions with the child	Using skills that promote positive parent–child interactions (e.g., demonstrating enthusiasm, following child’s interests, offering appropriate recreational options); providing positive attention
8.c	Responsiveness, sensitivity, nurturing	Responding sensitively to child’s emotional and psychological needs (e.g., soothing); providing developmentally appropriate physical contact and affection

^1^This taxonomy has been developed for use by other authors based on a number of leading reviews (Chorpita and Daleiden, 2009; Lee et al. 2014; Michie et al. 2013; Kaminski et al. 2008) on behavioral treatment techniques

## Motivational Accounts of ADHD Relevant to Behavioral Parent and Teacher Training for ADHD

For this review, theoretical accounts of ADHD relevant to the instrumental learning principles in BPT are those focusing on responses to reward and/or learning. Several theoretical accounts of ADHD incorporate altered processing of positive reinforcement as a possible causal mechanism underlying symptoms of ADHD (Luman et al. 2010). Three of these theories, the Dopamine Transfer Deficit [DTD] hypothesis (Tripp and Wickens 2008), the Dynamic Developmental Theory [DDT], (Sagvolden et al. 2005), and the dual pathway model (Sonuga-Barke et al. 2010, 2003, 2005) make explicit predictions about the learning and behavior of children with ADHD in response to positive reinforcement (relevant for the techniques of rewarding or attending to adaptive behavior in BPT). Douglas (Douglas 1989; Douglas and Parry 1994) separately proposes that children with ADHD have an underlying self-regulatory deficit manifest by abnormal responses to reward together with abnormalities in attention, inhibition, and arousal. She offers hypotheses about responses to reward and its failure to appear, thereby referring to Amsel’s frustration theory (Amsel 1958, 1962,1992a,1992b). Amsel (1992a) describes some specific predictions when applying his frustration theory to children with ADHD.

Both DTD and DDT focus on the neuromodulator dopamine, suggesting changes in dopamine signaling lead to altered sensitivity to positive reinforcement in children with ADHD. The dual pathway model acknowledges the role of dopamine in executive dysfunctions and delay aversion. Douglas’s work emphasizes both behavioral and emotional responses to reward. Some of these theories make predictions about extinction processes (relevant for negative punishment, e.g., the techniques of response cost, ignoring, time-out in BPT) of non-adaptive behavior and behavioral persistence of learned adaptive behavior), but none, with the exception of Amsel, make predictions about responses to positive punishment (e.g., the techniques of correction, overcorrection, natural consequences in BPT) in ADHD. Together these theories and their predictions may have implications for the techniques used in PBT with children with ADHD. These theories and their predictions are briefly reviewed below, with the predictions and the strength of the empirical evidence for them summarized in Table 2 below. We did not conduct a systematic review of the empirical evidence for every prediction, both authors have extensive knowledge and publications of the field of learning and ADHD and this was used as initial evidence. Additionally, we went forward and backwards in our searches using key papers; references and citations of key papers for every prediction were screened. In case of absence of evidence, specific searches were conducted for that topic.

### Dopamine Transfer Deficit Hypothesis

The DTD hypothesis (Tripp and Wickens 2008) assumes that in humans, as demonstrated in animals (Pan et al. 2005; Schultz 1998), there is an increase in dopamine cell firing (phasic dopamine release) in response to unexpected rewards. When a stimulus (cue) reliably precedes reward delivery the increased dopamine cell firing shifts from reward delivery to the reward-predicting cue, coming to serve as a conditioned reinforcer. This anticipatory dopamine firing is thought to deliver immediate and continuous reinforcement at the cellular level when behavioral reinforcement is delayed or discontinuous i.e., the conditioned cue serves to bridge delays between an action and reward delivery.

In children with ADHD, the transfer of the dopamine response to previously neutral cues is thought to be disrupted, leading to an absent or diminished anticipatory dopamine signal (i.e., impaired acquisition of conditioned reinforcers). As a result, when behavioral reinforcement is delayed or discontinuous dopamine signaling is also delayed or discontinuous resulting in ineffective reinforcement at the cellular level. Such impaired anticipatory dopamine firing is believed to negatively impact learning from reward and to disrupt control of behavior by its consequences in children with ADHD. The following predictions are made by the DTD. Children with ADHD are expected to demonstrate:

1. A stronger preference for immediate over delayed reinforcers
2. Poorer performance under partial or discontinuous schedules of reinforcement
3. Normal performance under continuous reinforcement schedules
4. A faster rate of extinction/more rapid extinction of unreinforced behaviors
5. A reduced or absent Partial Reinforcement Extinction Effect
6. Impaired acquisition of conditioned reinforcers which would lead to poorer stimulus control/ weaker control of behavior by reward-predicting cues
7. Increased sensitivity to the influence of individual occurrences of reinforcement, increasing the risk that non-target behaviors will be reinforced by other events.

### Dynamic Developmental Theory

Dopamine neurons normally fire at low tonic rates, showing phasic burst activity following a reward. The DDT (Sagvolden et al. 2005) proposes this tonic activity is reduced in children with ADHD (hypofunctioning dopamine systems) leading to stunted dopaminergic activity changes in response to unexpected reward (increased activity) and reward omission (decreased activity). These changes in dopamine functioning are predicted to lead to a steeper and shorter delay-of-reinforcement gradient. Under such a gradient the time window for linking reward-predicting stimuli (cues) or actions to reinforcement is shorter, i.e., a reinforcer loses its value more quickly as the delay between it and the desired behavior increases. As a consequence, children with ADHD are expected to:

1. Prefer immediate over delayed rewards
2. Learn more efficiently when rewards are immediate and frequent
3. Show more variable responding, with only short sequences of behavior reinforced
4. Demonstrate slower extinction (abnormally low tonic dopamine activity blunting phasic depression of tonic dopamine levels following reward omission)
5. Demonstrate slower establishment of conditioned reinforcers (necessitating higher value rewards to normalize the reinforcement process).

### Evidence for the DTD/DDT

With the exception of extinction, the predictions of DTD and DDT show substantial overlap, their purported neurobiological mechanisms involving different dysfunctions of the dopamine system. Evidence from human behavioral and imaging studies is available to support some, but not all of these predictions. There is consistent evidence that children with ADHD show a stronger preference for immediate over delayed reward compared with typically developing children (hypothesis 1 DTD/DDT). This has been demonstrated in choice delay tasks and temporal discounting tasks where children with ADHD are more likely to select small immediate over larger delayed rewards (see also evidence for the Dual Pathway Model below) (Marx et al. 2018; Patros et al. 2016). This has also been demonstrated using signal detection methodology where children with ADHD show a stronger preference (bias) for immediate over delayed reward when the total delay and size of rewards is the same across response alternatives (Tripp and Alsop 2001).

The evidence base comparing the performance of children with and without ADHD under schedules of partial or discontinuous reinforcement is limited and the findings are somewhat mixed (hypothesis 2 DTD/DDT). In their 2005 review, Luman and colleagues concluded there is some evidence for impaired performance in children with ADHD during intermittent compared with continuous reward delivery (Luman et al. 2005). Results from some studies suggest the performance of children with ADHD is more similar to that of controls under schedules of continuous or near continuous reinforcement (Douglas and Parry 1994; Freibergs and Douglas 1969; Parry and Douglas 1983) than under partial reinforcement (hypothesis 3 DTD/hypothesis 2 DDT). Furthermore, Aase and Sagvolden (2006) documented increased variability of responding under intermittent reinforcement in children with ADHD. On the other hand De Meyer and colleagues (De Meyer et al. 2019a) found no difference in the performance of children with ADHD and typically developing children under partial reinforcement conditions, the performance of both groups impaired under partial compared to continuous reinforcement, similar to an earlier report by Barber et al. (1996). Recent work by Luman and colleagues suggests impaired learning in those with ADHD under continuous and partial schedules of reward, especially in the initial stages of learning (Luman et al. 2020).

A small number of studies report on the performance of children with ADHD under conditions of extinction (Alsop et al. 2016; Cunningham and Knights 1978; De Meyer et al. 2019a; Douglas and Parry 1994; Furukawa et al. 2017a; Iaboni et al. 1997; Sagvolden et al. 1998) (hypothesis 4 DTD/ DDT). Alsop et al. (2016), De Meyer et al. (2019a), and Sagvolden et al. (1998) reported differential behavioral effects in children with ADHD during extinction following partial reinforcement. Alsop et al. (2016) reported typically developing children, but not those with ADHD, modified their response pattern during extinction. Similarly De Meyer et al. (2019a) reported children with ADHD were less likely to engage in other behaviors to try to obtain rewards once reward was discontinued. Both may indicate limited ability in those with ADHD to explore other behavioral options for reward following omission of an expected reward. Sagvolden et al. (1998) assessed rates of lever pressing in children with and without ADHD under alternating Fixed Interval (FI) and extinction schedules. They used a FI30s schedule, i.e., the first response after an interval of 30 s was rewarded. Responding in the ADHD group was elevated, compared with controls, under both schedules. Iaboni et al. (1997) reported children with ADHD failed to show an increase in skin conductance in response to removal of continuous reward, but no difference in their rate of responding compared with controls. Cunningham and Knights (1978) reported that typically developing children showed greater resistance to extinction (a higher percentage of correct responses) after punishment than hyperactive children, but no group differences following omission of reward. Only the study by Douglas and Parry (1994) directly assessed the speed of extinction, i.e., how quickly children ceased responding following omission of reinforcement. Given the option to stop responding after 10 (of 20) extinction trials, more children with ADHD choose to quit playing/responding, suggesting removal of reinforcement leads to more rapid cessation of previously rewarded actions in those with ADHD. This is important for BPT for ADHD as rewards are typically reduced, or phased out, following acquisition of adaptive behavior.

In sum, although only a few studies have directly assessed performance under extinction, most find some evidence of differential responding during extinction in those with ADHD (e.g., not engaging in other behaviors to try to obtain rewards); however, none specifically report slower extinction (as predicted by DDT). While Sagvolden et al. (1998) report higher rates of responding by children with ADHD during extinction, this may reflect overall increased response rates in this group. Only one study describes reduced persistence in ADHD (as predicted by DTD) (Douglas and Parry 1994). However, Luman et al. (2020) recently reported children with ADHD struggled more than typically developing [TD] children to apply previously learned knowledge when reward was omitted. They interpreted this as support for the DTD prediction of faster extinction in ADHD. Added to this, Sali and colleagues (Sali et al. 2018) provide preliminary evidence of faster extinction of prior learning in children with ADHD compared to TD controls in a study of value-driven attention capture.

The Partial Reinforcement Extinction Effect [PREE] is the phenomenon in which behavior acquired under conditions of partial reinforcement (i.e., using a less than 100% contingency between behavior and reinforcement) is more persistent under extinction than behavior acquired under continuous reinforcement (hypothesis 5 DTD). Only one study has explicitly tested the PREE in ADHD (De Meyer et al. 2019a), this paper shows intact PREE in children with ADHD when looking at the number of correct responses as the outcome. As the extinction period in this study was brief (2 min), it is not known if the PREE is maintained in children with ADHD under extended extinction conditions. Cunningham and Knights (1978) recorded performance under extinction in hyperactive and TD children following learning under continuous and partial reinforcement; however, interpretation of their data is difficult due to small sample sizes and large standard deviations.

Functional imaging studies offer evidence for impaired acquisition of conditioned reinforcers in ADHD (hypothesis 6 DTD/hypothesis 5 DDT). Several fMRI studies demonstrate reduced BOLD activity in the ventral striatum during reward anticipation (neural response to cues that predict reward) in those with ADHD compared with TD individuals (Furukawa et al. 2014; Plichta and Scheres 2014). These data would predict reduced behavioral responding to cues associated with reward in those with ADHD; however, such behavioral evidence for altered reward cue conditioning is not currently available.

Only one study has assessed whether the behavior of children with ADHD is influenced more by individual instances of reward than the behavior of controls (hypothesis 7 DTD). Using a signal detection task, Tripp and Alsop (1999) found that TD children showed a consistent preference for the response alternative that provided the higher rate of reinforcement, irrespective of the last reward received. In contrast, those with ADHD responded on the basis of the most recently received reward. This result implies that the behavior of children with ADHD is influenced less by their prior experiences of reward, i.e., their reward history, and is more susceptible to the last reinforcement received, providing initial evidence for hypothesis 7 of DTD. In practical terms this would increase the likelihood of non-target behaviors being reinforced in children with ADHD. Indirect evidence that the behavior of children with ADHD is influenced less by their history of reinforcement is reported by Sali et al. (2018). They demonstrated the responses of children with ADHD were slowed less by the presence of a previously reward-associated distractor than their TD peers.

Regarding the DDT prediction (hypothesis 3, DDT) that those with ADHD show more variable responding, there is consistent evidence that the responses of children with ADHD show increased variability compared to those of typically developing children across a range of tasks (Andreou et al. 2013; Kofler et al. 2013; Tamm et al. 2012), but how this relates to only short sequences of behavior being reinforced is unclear.

### Dual Pathway Model

The dual pathway model (Sonuga-Barke et al. 2010, 2002, 2003) proposes two complimentary “psycho-patho-physiological” pathways to ADHD: one involving executive dysfunction the other delay aversion. Here we focus on delay aversion given its link with reinforcement learning, BPT, and the focus of this paper. Behaviorally, delay aversion refers to the stronger preference of children with ADHD for small immediate over larger delayed rewards. This is hypothesized to arise from impairments in the neural signaling of delayed rewards leading to elevated levels of delayed reward discounting. For children with ADHD waiting and/or delayed reward acquires negative emotional significance following repeated pairing with disapproval or failure (in response to their failure to wait) from their environment (e.g., reactions of parents/teachers/peers), which leads to behavior designed to escape from or avoid delay, depending on situational constraints. Such behaviors will be maintained by negative reinforcement (i.e., escaping the negative feeling associated with waiting). According to the delay aversion theory children with ADHD will:

1. Show a negative emotional reaction to delay
2. Escape or avoid situations or actions that involve delay
3. Demonstrate a strong preference for immediate over delayed outcomes, where this choice is available
4. Attempt to modify the experience of waiting when escape or avoidance is not possible, i.e., engage in patterns of behavior that minimize the experience/perception of delay or that serve as, or result in, immediate reinforcement.

There is a substantial empirical evidence that children with ADHD exhibit increased sensitivity to reward delays together with a strong preference for immediate over delayed reward, in studies using choice paradigms, and that they act to escape or avoid situations that involve delay (Hypothesis 2 and 3) (Marx et al. 2018; Patros et al. 2016). There is much less evidence that they show a negative emotional reaction to delay. Imaging studies, with adolescents and adults show increased amygdala activation, a region known to be implicated in the processing of aversive events, in those with ADHD in response to delays (Lemiere et al. 2012; Mies et al. 2018; Van Dessel et al. 2018; Wilbertz et al. 2013). To the best of our knowledge the potentially altered emotional response to waiting has yet to be assessed behaviorally (Hypothesis 1), although there is initial behavioral evidence that individuals with ADHD show an attentional bias toward delay-related cues, which may suggest heightened emotional salience of delay (Sonuga-Barke et al. 2004). Anecdotally, parents report children with ADHD dislike waiting. Hypothesis 4 is supported by the results of a series of studies (Antrop et al. 2002, 2005,2000,2006; Bitsakou et al. 2006) that show that children with ADHD engage in more hyperactive behavior or increased rates of responding in delay tasks or waiting situations, except when stimulation is provided.

### ADHD as a Deficit in Self-regulation

Douglas proposes that children with ADHD have an underlying self-regulatory deficit that includes abnormal responses to reward (Douglas 1989; Douglas and Parry 1994). In analyzing the reactions of children with ADHD to reinforcement schedules she cautions against focusing exclusively on the incentive value of rewards. Referencing the learning theories of Amsel (1958, 1962), she emphasizes the importance of considering the emotional effects of both reward and non-reward, arguing researchers and clinicians should consider the possible negative consequences of some reward manipulations in children with ADHD. Specifically, the possible frustrating, distracting and arousing effects of different reinforcement schedules, including reactions to the failure of expected rewards to appear. Reviewing the work of her group on the effects of different reinforcement schedules on cognitive task performance Douglas (Douglas 1989; Douglas and Parry 1994) hypothesized that children with ADHD:

1. Have an abnormally strong inclination to seek immediate reward
2. Are unusually vulnerable to the possible arousing and distracting effects of reward
3. Become abnormally frustrated when anticipated rewards fail to appear.

As noted above there is substantial empirical evidence that children with ADHD show a heightened tendency to seek immediate reward (Hypothesis 1). We identified only two papers that provide evidence of increased frustration in children with ADHD when anticipated rewards fail to appear; assessed by facial responsivity (Wigal et al. 1998) or the force of lever pulling (Douglas and Parry 1994) (Hypothesis 3). There is limited evidence for hypothesis 2, i.e., increased vulnerability to the arousing and distracting effects of reward. One study explicitly tested distraction by reward in ADHD, and showed children with ADHD were significantly *less* distracted by stimuli previously associated with reward compared to TD controls (Sali et al. 2018). Luman and colleagues (Luman et al, 2009) reported the performance of children with ADHD, but not that of TD controls deteriorated on a time estimation task under both reward and penalty conditions, leading them to conclude the children may have been *more* distracted by reinforcement stimuli. In contrast to this finding, several studies report reward improves cognitive task performance in those with ADHD, including enhanced vigilance and sustained attention (e.g., Bubnik et al. 2016; Dovis et al. 2012; Fosco et al. 2015). On balance, there seems to be little evidence for increased vulnerability to the arousing and distracting effects of rewards in ADHD.

### Frustration Theory

According to Amsel’s frustration theory (Amsel 1958, 1962, 1992a, 1992b), based on prior rewarded behavior, individuals develop expectancies that such behavior will be rewarded in future. However, when reward is not scheduled, for example under partial reinforcement schedules, or during extinction, individuals experience an emotional response, referred to by Amsel as “primary frustration”. During future occurrences of the behavior, this experience led to an expectancy for frustration (Amsel’s “anticipatory frustration”). Subsequently, individuals experience both expectancies for reward and non-reward, which results in an approach-avoidance conflict. For learning and behavioral persistence to occur individuals must learn to continue responding/displaying adaptive behavior while experiencing this approach-avoidance conflict and anticipatory frustration, i.e., develop frustration tolerance, which not all individuals are able to do. This increased frustration may lead to paradoxical effects, and an increased response to non-reward cues, which displays itself in increased motor activity (e.g., hyperactive behavior). Amsel (1992a) acknowledges that he generally concurs with Douglas’s view but makes some more specific predictions regarding how his theory would apply to children with ADHD (Amsel 1992a):

1. With intermittent reinforcement or punishments of other kinds children with ADHD do not persist as “frustration tolerance” is not achieved
2. As a result of this prolonged frustration during partial reinforcement they (children with ADHD) have a reduced tendency to persist in the face of non-reward (reduced behavioral persistence in extinction)
3. Hyperactivity in ADHD is due to increased responsiveness to stimulation from anticipatory frustration in partial reinforcement conditions.

As noted earlier only two papers describe evidence for a heightened frustrative response in children with ADHD during intermittent reinforcement (Douglas and Parry 1994; Wigal et al. 1998) (Hypothesis 1). The paper by Wigal et al. (1998), provides preliminary evidence of increased frustration, and its effects on behavioral persistence, under intermittent reinforcement conditions in children with ADHD (Hypothesis 2). The causal link between frustration and hyperactivity (Hypothesis 3) has never been explicitly tested. In general detailed analysis of frustration and learning under conditions of partial reinforcement and extinction has not been undertaken. There is more indirect evidence from a questionnaire study that children with ADHD experience greater frustration in response to uncertainty (as in partial reinforcement conditions) (Gramszlo et al. 2018) and evidence that they are more easily frustrated and quit earlier in challenging frustrative tasks than controls (Scime and Norvelitis 2006). However, to date the link with learning is not explored.

### Summary of Evidence of Motivational Accounts of ADHD

Above we reviewed five motivational accounts addressing the responsiveness of children with ADHD to reinforcement. All offer specific predictions regarding the behavior of children with ADHD in response to the delivery or none delivery of positive reinforcement and cues that signal reward. There is overlap in the predictions across theories, but not necessarily in the proposed mechanisms. A head-to-head comparison of the support for motivational accounts is complicated due to the different number of predictions and variability in the quality of the available evidence. The true value of these five theories lies is their ability to generate empirical studies to test their predictions/proposed mechanisms. In considering how BPT could be modified to better meet the needs of children with ADHD, a review of the strength of evidence for each prediction of every account may be more helpful. This information is provided in Table 2 below.

**Table 2:** Predictions/findings of altered reinforcement sensitivity in ADHD, expected impact on child behavior, and recommended practices for inclusion in behavioral parent and teacher training programs

Prediction/Finding^a^	Strength of evidence^b,c^	Theories^d^	Potential impact on child’s behavior	Recommendations for management
Positive reinforcement				
Prefer immediate over delayed reward	Very strong	DTD [1] DDT [1] Delay aversion [3] Douglas [1]	Act to avoid or minimize delay Impulsivity	Reinforce immediately where possible Educate caregivers to avoid/reduce experience of delay for children with ADHD Educate caregivers children may act to avoid/reduce delay resulting in impulsive behavior Link rewards to actions before and when delivering reward Teach older children how they can manage delay and self-reward Enhance the saliency of the expected behavior before and during delay-related situations (e.g., clear instructions, pictures of expected behavior), if possible reduce saliency of incidental immediate rewards in the child’s environment (e.g., remove distractors)
Escape/avoid situations that involve delay	Very strong	Delay aversion [2]	Impulsivity	
Impaired acquisition of conditioned reinforcers/impaired response to reward-predicting cues			More distractible off-task behavior Poorer stimulus control Poorer maintenance of behavior by delayed or discontinuous reinforcement	Draw child’s attention to rules/expectations of the current context and when these change Increase saliency of cues Scaffold/shape learning of adaptive behavior Highlight the outcome of the child’s behavior to them
Imaging studies	Very strong	DTD [6]		
Behavioral studies	None	DDT [5]		
Show more variable responding, with only short sequences of behavior reinforced				
More variable responding	Very strong	DDT [3]	More variable task performance	Maintain high rates of immediate reinforcement throughout tasks
Short sequences of behavior reinforced	None			
No performance deficit under continuous reinforcement Poorer performance under partial reinforcement	Limited (3) Limited/mixed (4/2)	DTD [3,2] DDT [2]	Learn less/more slowly under partial reinforcement Slower adaption of behavior to new situations/contingencies under partial reinforcement	Maintain high rates of reward during learning of new behaviors/skills (acquisition) Institute individualized stretching the ratio’s (gradually shift from more continuous to partial schedules of reinforcement) to install persistence of learned behavior. In doing so ensure rates of reinforcement are sufficient to maintain interest in a task/activity
Increased vulnerability to possible arousing and distracting effects of reward	Limited/mixed (1/1)	Douglas [2]	Reward may not be as effective as expected	Monitor responses to reward, revise reinforcement schedules as required over time
Increased influence of individual/recent occurrences of reinforcement on behavior	Limited (1 direct) (1 indirect)	DTD [7]	Increased risk of non-target behaviors being reinforced by other events/incidental rewards More off-task behavior due to increased distractibility toward incidental rewards Stronger environmental & weaker internal control of behavior	Educate caregivers of children’s vulnerability to incidental rewards, increasing the probability of non-adaptive behaviors being reinforced Remind children of setting specific rules Increase the saliency of discriminative stimuli to reduce the impact of incidental rewards Reduce distracting/competing recent rewards by increasing environmental and temporal structure
Faster extinction	Limited (1 + 2 indirect)	DTD [4] Non-specific	Less behavioral persistence Learn/demonstrate less new exploratory/adaptive behavior in the context of refraining from formerly rewarded behavior	Use of stretching the ratio’s to install behavioral persistence Don’t assume children will “know what to do” when you stop rewarding a learned behavior. Remind children of the expected adaptive behavior once extinction starts, i.e., when reward for adaptive behavior stops or is reduced in frequency For older children teach them to self-reinforce under conditions of extinction
*Differential extinction effects*	Limited/mixed (3/2)			
Heightened emotional response when anticipated rewards fail to appear	Limited (2)	Douglas [3] Amsel [1]	More frustration in daily life (continuous reinforcement in daily life is rare)	Strive for consistency of rewards as much as possible Educate caregivers about emotional effects of reinforcement manipulations (e.g., response cost/ ignoring/ partial reinforcement) Some children may need to be taught emotion regulation techniques
Reduced persistence of behavior in response to partial reinforcement/punishment/extinction due to prolonged frustration	Limited (1 direct) (1 indirect)	Amsel [2]	Higher levels of frustration in daily life interfere with learning persistent adaptive behavior	Educate parents about frustrative effects of non-reward Teach frustration management techniques to parents/children Stretching the ratio’s to gradually expose child to frustration and install persistent adaptive behavior
Additional relevant findings				
*Poorer matching of behavior to reinforcement contingencies*	Moderate/mixed (5/2)	Non-specific	Poorer/slower adaptation of behavior to changing environmental expectations	Reduce behavior-consequence ambiguity: Explicitly inform children of expectations/rules ahead of time Explicitly inform child when rules/ contingencies change Institute overlearning Provide sufficient opportunity to transition when rules/requirements change Reinforce desired behavior going into new situations Educate caregivers of children’s difficulty modulating behavior, especially when/if rules/expectations are not made explicit
*Failure to adapt behavior to situational demands (when there is a delay)*	Limited (1)	Non-specific (CDL)	Environment does not serve as a cue for adaptive behavior	Increase the saliency of the situation and the situation-response association Use Differential Outcomes, i.e., apply different reinforcers for different adaptive behaviors/skills
Negative punishment				
*Mild punishment (i.e., response cost) improves on-task behavior and performance*	Strong/mixed (11/3)	Non-specific	Reduces undesirable behavior in the short-term, long-term effects unclear Potentially more errors under threat of punishment	Educate caregivers of potential negative side-effects Use cautiously Avoid extended use Monitor the emotional response of children to negative punishment Reward alternative adaptive behaviors as an alternative to use of punishment Some children may benefit from learning emotion regulation techniques
Mild punishment (e.g., ignoring/ failure to deliver rewards) may	Limited	Amsel [1]	Increased emotionality to ignoring/non-delivery of rewards Faster reduction/stopping of learned adaptive behavior when no reward follows	
Increase frustration	(2 + 1 indirect)			
Reduce behavioral persistence	(1)			
Positive punishment				
*Positive punishment causes unwanted side-effects in ADHD*	Limited (3)	Non-specific	More errors on tasks Missed learning opportunities (resulting from efforts to avoid punishment) Lack of persistence in activities perceived as punishing (including tasks experienced as frustrating and/or effortful) Slower responding in punishment situations	Educate caregivers about negative side-effects of punishment Avoid use wherever possible Use all the other recommendations to prevent use of punishment (make rules/ consequences explicit/ provide non-punitive calm reminders/ reinforce adaptive behavior)
Not currently supported				
Diminished PREE	None	DTD [5]		
Slower extinction	None	DDT [4]		
Hyperactivity is due to increased responsiveness to stimulation from anticipatory frustration in partial reinforcement conditions	None	Amsel [3]		

^a^Findings not linked to a specific theory are presented in italics

^b^Strength of evidence based on the literature reviewed in the manuscript: Very strong = multiple empirical studies in support of the prediction/finding, including at least one published meta-analysis; Strong = more than 10 empirical studies available in support of the prediction/finding; Moderate = five to 10 empirical studies available in support of the prediction/finding, number of studies in brackets; Limited = less than five empirical studies available in support of the prediction/finding, number of studies in brackets; Mixed = some empirical studies available whose data counters the prediction/finding, number of studies in brackets (support/counter); None = currently no evidence available to support the prediction

^c^Strength of evidence here refers to the number of available studies, not the quality of the studies or sample sizes

^d^Numbers in square brackets reflect prediction numbers in the text; Non-specific indicates empirical findings not linked to specific theories

The research reviewed indicates there is *some* evidence to support all but one of the predictions arising from both DTD (no evidence for a reduced/absent PREE) and DDT (no evidence for slower extinction). The delay aversion hypothesis makes fewer predictions than either DTD or DDT and support for all but one of its predictions is strong to very strong. The exception being that children with ADHD show a negative emotional reaction to delay, behavioral evidence for this prediction is to date limited. Douglas and Amsel’s theories both focus on the emotional impact of reward and non-reward. There is very strong evidence for Douglas’s proposal that children with ADHD have an abnormally strong inclination to seek immediate reward. The evidence for her predictions that children with ADHD are more vulnerable to the distracting effects of reward and to increased frustration in face of non-reward is limited. Similarly, there is limited evidence for Amsel’s predictions of increased frustration and reduced behavioral persistence during intermittent reinforcement. To date Amsel’s hypothesized causal link between frustration and hyperactivity has not been tested. In sum, currently the strength of evidence favors the delay aversion hypothesis, in part reflecting the many studies that have tested its predictions as opposed to fewer studies testing the specific predictions of the other theories. However, to varying degrees, the other theories offer explanations for the occurrence of delay aversion in children with ADHD. More research is clearly needed to evaluate the predictions of these other theories. Recognition of their importance to the ongoing development of BPT may encourage this research.

## Additional Motivational Research Relevant to the Use of Operant Techniques in BPT

### Responses to Punishment in Children with ADHD

Compared to reward, the behavioral sensitivity of children with ADHD to punishment has received limited theoretical and empirical attention. Only Amsel (1992a) makes a specific prediction regarding responses to punishment in ADHD, proposing that punishment is more frustrating to children with ADHD and causes a lack of behavioral persistence in those with ADHD.

Experimentally, mild punishment, operationalized in these studies as negative punishment (response cost), has been shown to *enhance* the performance of children with ADHD across a range cognitive tasks^2^; in just under half of these studies response cost improved task performance in children with ADHD only, although one cannot rule out that this is due to ceiling effects in performance among control group participants (Carlson et al. 2000; Carlson and Tamm 2000; Iaboni et al. 1995; Slusarek et al. 2001). In the other studies no difference was found in the performance of children with ADHD and controls under conditions of response cost (Crone et al. 2003; Cunningham and Knights 1978; Firestone and Douglas 1975; Groen et al. 2013; Solanto 1990). Two studies did show children with ADHD were less accurate than controls under the threat of response cost (Crone et al. 2003; Luman et al. 2009).

With regards to the effects of negative punishment on everyday off-task behavior, an early case report study showed response cost was effective in maintaining on-task behavior (Rapport et al. 1982). Other types of punishment have received limited attention: a case series study (*n* = 8 “hyperactive” boys) showed that negative punishment (in this case ignoring) was ineffective in reducing off-task behavior, leading the authors to conclude that behaviors not driven by attention should not be managed by ignoring (Rosén et al. 1984). In a summer treatment program setting one study found time-out to be effective in reducing non-compliant, destructive and aggressive behavior in children with ADHD (Fabiano et al. 2004).

Two studies have assessed the effect of positive punishment on on-task behavior in ADHD. Rosén et al. (1984) showed that instituting positive punishment (mostly verbal reprimands), without positive reinforcement was effective in decreasing high levels of off-task behavior. Another study showed positive effects of punishment (honking a horn in response to periods of off-task behavior) for on-task behavior in children with ADHD compared to controls; however, children with ADHD made more errors under threat of punishment (Worland 1976). Two recent studies demonstrated a differential response to punishment (a combination of positive punishment [laughing sound] and negative punishment [response cost/loss of points] in children with ADHD on a response allocation task Furukawa et al. 2017a, 2019b). Compared with typically developing controls, children with ADHD showed greater sensitivity to punishment evidenced by an increase in response bias toward the less punished response alternative (over trials) together with slower responding and more response switches (less persistence on the more punished alternative) following instances of punishment. This pattern of responding was not advantageous overall, the ADHD group accumulating fewer points than controls (Furukawa et al. 2017a, 2019b).

Taken together these results suggest that mild punishment in the form of response cost/time-out (negative punishment) may increase on-task behavior and reduce undesirable behaviors effectively in the short-term. However, its long-term use and/or the use of positive punishment may have unanticipated and unwanted side-effects in children with ADHD when used to manage or shape their behavior. They may focus more on avoiding punishment, it may lead to reduced accuracy, or decrease their engagement in desirable behavior that would lead to better outcomes. Amsel’s hypothesis that punishment led to reduced persistence in children with ADHD was supported by two studies showing a lack of behavioral persistence on the response alternative associated with the higher rate of punishment (Furukawa et al. 2017a, 2019b). However, a causal link between frustration and lack of persistence is not clear from these findings.

### Learning What Behavior is Appropriate in Which Situation: Conditional Discrimination Learning

Adaptive behavior requires the constant adjustment of one’s responses to different situational demands, this is also called conditional discrimination learning (a form of instrumental associative learning; learning what behavior to perform in which situation) (Martínez et al. 2009). A recent study by De Meyer et al. (2019b) shows that this learning is intact in ADHD when there is no delay between the situational information and the required behavior. However, once a short delay is inserted between the situational cue and the required response, which is common in daily life, children with ADHD are less able to learn the required/adaptive behavior than typically developing controls.

### Matching Behavior to Available Reinforcement Contingencies

Behavior management programs teach parents to reward instances of appropriate behavior as a means of increasing the likelihood these actions will be repeated. The assumption being that children adapt their response allocation to reinforcer availability. Given the evidence that children with ADHD have an altered sensitivity to reinforcement, the extent to which their behavior tracks the reinforcement contingencies operating is an important empirical question.

A small number of studies have compared the response allocation of children with and without ADHD using concurrent variable interval scales (VI/VI; Kollins et al. 1997; Taylor et al. 2010); reversal learning tasks (Chantiluke et al. 2015; Hauser et al. 2014) and signal detection tasks (Alsop et al. 2016; Furukawa et al. 2019a). Kollins et al (1997) and Talyor et al. (2010) reported the behavior of children with ADHD does not match the contingencies in operation as well as that of typically developing children. Cross-culturally children with ADHD adjust their behavioral responses to changing reinforcer availability less efficiently than their typically developing peers (Alsop et al. 2016; Furukawa et al. 2017b, 2019a). These latter deficits are most apparent when rates of reinforcement are relatively low and the contingencies are not made explicit. Chantiluke et al (2015) and Hauser et al (2014) reported intact reversal learning in children with ADHD under conditions of frequent reinforcement. On balance the available research indicates children with ADHD are less efficient in matching their response allocation to reinforcer availability than typically developing children.

### Responses to Remediation Strategies for Learning Deficits in Children with ADHD: Stretching the Ratio’s and Differential Outcomes

As ADHD is considered to be characterized by partial reinforcement learning deficits, some of the above theories hypothesize that the partial reinforcement extinction effect [PREE, see above] may be disturbed in ADHD (Amsel 1992a, b, c; Tripp and Wickens 2008). To overcome these proposed PREE deficits and improve generalized adaptive behavior in ADHD, strategies to enhance behavioral persistence in ADHD need to be explored. One such strategy is the implementation of a stretching the ratio’s procedure, in which the level of reinforcement during acquisition is gradually reduced from continuous to partial. This procedure may also be relevant for improving long-term effects of behavioral contingency programs. Only two studies have explored this procedure in children with ADHD (Barkley et al. 1980; De Meyer et al. 2019b). Both studies showed positive effects of introducing a gradual reduction in reinforcement rates for appropriate behavior and highlight the potential of this technique for use with children with ADHD as well as typically developing children.

As noted earlier, children with ADHD have difficulty adapting their behavior to different environmental demands (De Meyer et al. 2019b; Nigg and Casey 2005; Sagvolden et al. 2005, b). One potential solution to this difficulty is the use of Differential Outcomes [DO]. Differential outcomes are a procedure in which specific stimulus–response relations are reinforced using response-unique, rather than general, outcomes (Martínez et al. 2013; Urcuioli 2005). This may be operationalized as giving specific reinforcers for various forms of situationally adaptive behavior instead of one generic reward (as often used in contingency management programs). This may be operationalized, for example, by giving a blue sticker for getting your book ready in math class and a yellow sticker for showing on-task behavior during a reading class. The idea behind this is that the associative structure of the stimulus–response is strengthened. The child forms not only a stimulus response association but also a specific stimulus outcome association. There is evidence for the effectiveness of this procedure in individuals with range of disorders, e.g., Alzheimer’s, Prader–Willi, Autism and Korsakoff (Esteban et al. 2014; Hochhalter and Joseph 2001). To date only one study describes the effectiveness of DO with children with ADHD (De Meyer et al. 2020). This study shows that providing differential outcomes, as compared to generic non-differential outcomes, improves the ability of children with ADHD to learn stimulus response associations to the same level as typically developing children.

## ADHD Specific Adaptations to Behavioral Parent and Teacher Training Programs Based on Empirical Evidence

Based on the empirical evidence for altered reinforcement sensitivity in ADHD reviewed above, we describe implications for and potential adaptations to BPT training programs for ADHD, thereby making a distinction between recommendations for the core elements of BPT programs described before (psycho-education, operant/instrumental learning, pavlovian conditioning, stimulus control techniques). These treatment recommendations are theoretically and logically derived and informed by clinical practice. Some of these recommendations may already be in use by experienced clinicians in their work with parents and teachers managing ADHD. Other recommendations may be new to clinicians, parents and teachers (e.g., use of stretching the ratio’s or differential outcomes). Additionally, in Table 2 we present the predictions from the five theories and other relevant findings, quantify the strength of evidence supporting them, outline how these learning deficits would translate into the behavior of children with ADHD, and offer specific management recommendations for these behaviors. Presented this way we are able highlight the links between deficits in learning from reinforcement in ADHD, symptoms of ADHD, and the theoretically and logically derived treatment recommendations. Within each section of the table the predictions/findings are ordered by the strength of the evidence supporting them.

### Psycho-education

There is accumulating evidence that children with ADHD differ from typically developing children in their processing of motivationally significant events. Parents, teachers and clinicians need to understand these differences to effectively adapt their use and teaching of behavior management principals. This is important to both the successful implementation of behavior management techniques and to the parent/teacher–child relationship. Understanding why the reactions/behavior of children with ADHD to consequences and situational cues differs from that of other children will reduce adult frustration and enhance their motivation to persist with BPT. In addition to advising involved parents and teachers that children with ADHD differ in their responsiveness to consequences, the following information should be shared: (1) Children with ADHD may act to avoid/reduce delays (this recommendation has the strongest empirical support); (2) be more upset/frustrated when expected rewards are not forthcoming; (3) be more vulnerable to incidental/accidental rewards in the environment (potentially increasing the likelihood of non-adaptive behaviors being reinforced); (4) give up more easily when tasks are perceived as too difficult or punishing; (5) struggle to understand what is expected of them if rules/expectations are not made explicit.

### Instrumental Learning

Rewards and punishments are defined by their actions on the behaviors that follow them, i.e., behavior that is rewarded increases in frequency while behavior that is punished is expected to decrease in frequency. The literature reviewed indicates children with ADHD differ from TD children in their response to the timing and density of reinforcement. Based on these data we recommend in BPT for ADHD: (1) to maintain high rates of immediate reinforcement during the learning of new behaviors/skills (strong empirical support); (2) to consider instituting individualized stretching of the ratios, i.e., gradual reduction in the density of reinforcement, once new behaviors are acquired. This requires careful attention to the effects of reducing reward on the child’s behavior to ensure rates of reinforcement are sufficient to maintain interest in a task or activity, i.e., update functional analyses. Stretching the ratios offers an opportunity to gradually expose the child to the frustration of not getting a reward and installs persistent adaptive behavior; (3) to identify creative means to maintain high rates of immediate reinforcement throughout tasks or activities to reduce response variability and efforts to escape from, or modify, the experience of delay (strong empirical support); (4) to consider the use of differential outcomes (response specific reinforcers), which may increase the learning of situation-specific stimulus–response associations (for example differently colored tokens for various forms of situationally appropriate behaviors).

With regards to the use of punishment procedures in BPT, there is good evidence that mild punishment, e.g., response cost improves on-task behavior in children with ADHD. However, recent research, reviewed above, indicates that mild punishment can lead to more errors on tasks and increased emotionality. There is also evidence suggesting children with ADHD are *more* sensitive to punishment than TD children. For children with ADHD positive punishment leads to more errors, missed learning opportunities, and lack of task persistence. All of which argues strongly for caution in the use of punishment, especially positive punishment, with children with ADHD. Therefore it is recommended that (5) parents and teachers should, as much as possible, be encouraged to reward alternative adaptive behaviors to reduce the need to use punishment with children with ADHD. If the use of punishment is unavoidable, its extended use should be avoided and the emotional response of the child carefully monitored.

### Pavlovian/Classical Conditioning

As highlighted in the introduction, it can be difficult to separate the effects of instrumental learning and classical or Pavlovian conditioning on children’s behavior and learning. Both the DTD and DDT theories propose that children with ADHD show impaired acquisition of conditioned reinforcers. The DTD hypothesis suggesting this leads weaker control of behavior by reward-predicting cues (i.e., stimulus control). Recommendations to strengthen stimulus control are detailed below.

### Stimulus Control

Stimulus control techniques are important aspects of BPT programs. Commonly recommended practices including the provision of clear instructions and rules and the provision of structure are clearly important for children with ADHD. In addition we recommend parents and teachers be taught to: (1) explicitly draw the child’s attention to rules/expectations in their current context, i.e., what is expected of them, and *importantly* when these expectations change; (2) highlight the outcome of the child’s behavior to them to reduce behavior-consequence ambiguity. Linking rewards to actions before and when delivering reward will assist in scaffolding/shaping the learning of new behaviors; (3) increase the saliency of cues/discriminative stimuli to reduce the impact of incidental rewards, this is especially important in situations involving delay. Wherever possible reduce the salience of immediate incidental rewards, i.e., remove distractors, this may be achieved by further increasing environmental and temporal structure; (4) remind children of expected adaptive behavior when the frequency of reward is reduced. It is important not to assume children with ADHD will know what to do when a learned behavior is no longer rewarded.

### Self-management

In addition to providing parents and teachers with the skills to effectively implement behavior management techniques we suggest the outcomes of BPT can be enhanced with the introduction of specific skills training for children with ADHD, and possibly parents and teachers dealing with ADHD. Specifically we suggest clinicians consider the need to teach emotion regulation techniques/frustration management skills to children with ADHD and their parents. We also suggest consideration be given to teaching older children with ADHD how to manage the experience of delay, possibly through learning strategies of self-reward (strong empirical support). Self-reinforcement may also be helpful under conditions of extinction (reinforcement is absent) or when the reinforcement rates are less dense.

In sum, clearly there are varying levels of support for the recommendations offered, as indicated in Table 2. The strongest empirical evidence indicates children with ADHD prefer immediate over delayed reward and act to reduce the experience of delay. In daily life, however, it is unrealistic to immediately reward every instance of a desired behavior, necessitating the use of other procedures to help manage the behavior of children with ADHD. For this reason, we have not specifically ranked our recommendations, although we do indicate those for which strong empirical support is available and we state more tentative adaptations for those recommendations with less evidence. All of the adaptations proposed are very likely to be non-harmful and would likely benefit many other children whose parents and teachers participate in BPT. Furthermore, the currently limited evidence base supporting some of our recommendations reflects the absence of relevant research in the field.

## Discussion

Behavioral Parent and Teacher Training is an effective treatment with moderate effects in reducing objectively and subjectively measured behavioral problems and in enhancing parenting practices. However, for symptoms of ADHD the effects are somewhat less pronounced, i.e., effects are not corroborated by objective measures (Daley et al. 2014), and over time effects of training tend to dissipate (Lee et al. 2012). Clearly there is room for improvement in behavioral training for ADHD. Tailoring treatments to address reinforcement related deficits in ADHD may enhance the effects of BPT. There is a growing evidence base indicating that, as a group, children with ADHD differ in their “sensitivity” to reinforcement contingencies (Fosco et al. 2015; Luman et al. 2005, 2010). We argue that these differences should be considered and incorporated into the use of operant techniques in the behavioral treatment of children with ADHD [see Fig. 1 for rationale].

Based on a selective review of reinforcement-based theories of ADHD, their predictions, the empirical research testing these predictions, and other relevant research we provide an “update’ on how instrumental learning principals can be applied in behavioral treatment with families and teachers of children with ADHD. Many of these suggestions are consistent with current clinical practices (e.g., the use of immediate reinforcement), others, including the use of punishment with children with ADHD, offer new insights, practical suggestions for application, and some cautions. We also propose the use of remediation strategies for proposed deficits in learning not commonly used in BPT programs, e.g., the use of stretching the ratios to help with adaptation to partial reinforcement and enhance persistence of behavior, and the use of differential outcomes to strengthen stimulus response outcome associations and the learning of situationally appropriate behavioral responses.

The identification of alterations in the responses/reactions of children with ADHD to reinforcement contingencies is empirically based. The impact of these alterations on their daily and classroom behavior are logically derived from the results of these studies and informed by clinical experience. Practice recommendations are also logically and theoretically derived and, in some cases, e.g., stretching the ratios and differential outcomes, based on recent empirical studies. Additional research is required to evaluate the usefulness of many of the proposed modifications and/or additions to the arsenal of behavioral techniques, beginning for example with a series of microtrials thereby testing the use of specific techniques (e.g., a reward system with and without differential outcomes/ a reward system with and without stretching the ratio’s) with samples of parents of children with ADHD in more ecologically valid ways with real-life problem behavior. Microtrials enable testing of intervention components on immediate outcomes and thus seem relevant here (Howe et al. 2010; Leijten et al. 2015). Although in other clinical groups techniques proposed here such as differential outcomes have been used to train real-life adaptive stimulus response associations [for example in individuals with intellectual disabilities (Estevez et al. 2003); in individuals with Autism Spectrum Disorders (McCormack et al. 2017)] translating procedures such as differential outcomes to feasible easy to implement techniques for parents and teachers is a premise for effective use. In addition to determining the efficacy of these modifications such as differential outcomes in microtrials, the feasibility of these approaches for parents and teachers needs to be assessed and balanced with their effectiveness. If these techniques are proven effective and feasible in these microtrials, specific adaptations can be made to treatment manuals for children with ADHD.

Rather than repeat our practice recommendations from Table 2 and the specific adaptations described, here we address how they can be transferred to clinical practice. First, practitioners and researchers working with children and families dealing with ADHD will attest to the heterogeneity of ADHD. In making these recommendations we are not suggesting that all strategies need to be applied with all children. Some tailoring will be necessary to meet the needs of individual children with ADHD and their families. In the case of group delivered parenting programs this could take the form of specific modules for specific impairments, i.e., a core set of strategies, to which specialized recommendations, based on a detailed behavioral assessment of the child, could be added. Clinicians providing training to parents and teachers need to carry out topographical (a detailed specific and factual analysis) and functional analyses of problem behavior, it's antecedents and consequences, to determine the most appropriate set of behavioral interventions for each child and family, possibly varying across settings and contexts (e.g., it may be that one child has particular problems with the loss of anticipated rewards (frustration), while another child may fail to adapt behavior to situational demands). For the continued effectiveness of BPT beyond the training period, clinicians should also consider instructing parents and teachers how to carry out a topographical analysis for the effective management of future behavioral problems. Theoretically, alterations in reinforcement processing would be expected to destabilize ongoing adaptive behavior, necessitating regular monitoring and updating of behavior management of the child. For example, delays in reward lead to increased impulsivity and hyperactivity, heightened sensitivity to recent rewards to incidental reinforcement of maladaptive behaviors, and impaired acquisition of conditioned reinforcers to poor stimulus control of behavior.

Second, the delivery of behavioral interventions for children with ADHD relies on parents and teachers as the agents of change. To be effective in this role these individuals need to understand the nature of ADHD, and of particular importance here, how children with ADHD differ in their processing of reinforcement contingencies (i.e., psycho-education on reinforcement learning deficits in ADHD). Without such understanding parents and teachers may assume deliberate misbehavior on the part of the child, behavior which is better understood in terms of differential sensitivity to the contingencies operating in their environment. Such knowledge will help ensure the selection and implementation of the most appropriate management strategies, improved child behavior, and increased caregiver engagement. Furthermore, some parents may have ADHD themselves, and potentially similar altered reinforcement processing as their child (Furukawa et al. 2014; Marx et al. 2010; Plichta and Scheres 2014). Therapists should acknowledge this and take it into account when educating parents about ADHD and altered reinforcement sensitivity and when teaching them behavioral techniques. The same recommendations may apply to therapists teaching parents, as for parents teaching their children. For example, when parents with ADHD perceive the application of behavioral techniques as effortful, long, and unrewarding, this may lead to frustration and a lack of persistence in following through. Parents may benefit from the same recommendations as offered for children, for example being taught how to handle their own frustration or providing self-rewards for engaging in BPT.

Third, although to date there is minimal empirical support (Table 2), it is important for clinicians and caregivers alike to be aware that some children with ADHD may show increased emotionality to changes in reinforcement contingencies, i.e., removal of reinforcement (e.g., response cost), to positive punishment or to discontinuous (partial or non-contingent reinforcement) schedules of reinforcement. Clinicians and caregivers need to be mindful of this in interpreting children’s behavioral responses and also in their own choice of disciplinary strategies. In the short-term, negative consequences may led to improved behavior (e.g., time on-task), longer term they may lead to increased emotionality, more errors, less learning opportunities, and avoidance of effortful tasks. Although not tested empirically, clinically this heightened emotional reactivity is often observed (Graziano and Garcia 2016), which has recently led to the development of ADHD-specific emotion regulation treatments (Rosen et al. 2019), and these may be indicated for some children with ADHD for managing the frustration of failing to receive anticipated rewards.

Lastly, remediation techniques such as stretching the ratio’s and differential outcomes seem to work similarly in children with ADHD and typically developing children. Although more evidence is needed, with microtrials seeming to be promising ways to gather this evidence, training clinicians in the use of these techniques and having them implement them in BPT programs would seem warranted.

The number of studies addressing aspects of reinforcement sensitivity has been steadily increasing, many of which test specific theories or predictions about such sensitivity. However, this literature can be overwhelming to review. In an effort to organize this information we present the available literature in terms of whether or not it supports these theories and their predictions. Although we believe we have captured most of the relevant literature, the aim of the current review was not be a systematic review, thus it is possible that we have missed some evidence for a specific hypothesis. We see our effort as a first step in bridging the science to practice gap, by combining and translating empirical evidence of altered reinforcement sensitivity into clinical recommendations for behavioral parent training. To better fill this gap, additional steps may be needed, e.g., use more ecologically valid study designs and perhaps a more systematic review of the evidence of specific reinforcement sensitivity in ADHD. Given the lack of evidence for some predictions, a number of our recommended additions to behavior therapy are theoretically and logically derived, rather than extensively empirically tested.

It is clear that additional research is required to more fully evaluate the predictions regarding the responsiveness of children with ADHD to reinforcement. As much as possible, this research should explicitly test the predictions of the theories presented. It became clear, while preparing this manuscript, that the majority of research has focused on the behavioral sensitivity of children with ADHD to reinforcement contingencies, i.e., the effects of reward and punishment on-task performance or response choices (e.g., Bubnik et al. 2016; Demurie et al. 2012; Dovis et al. 2012). There is a shortage of research addressing the learning of new skills/behavior in children with ADHD and whether they show a differential response to reinforcement contingencies in this context. We are also limited in what is known about the emotional effects of different reinforcement manipulations in children with and without ADHD. Douglas, Amsel and Sonuga-Barke all propose that children with ADHD show strong negative emotional reactions to delays and to failures in the delivery of anticipated rewards. While anecdotal reports support these hypotheses, empirical behavioral evidence is sparse. Preliminary evidence supports the use of remediation strategies (e.g., stretching the ratios and differential outcomes), but more research and replication is needed.

We are confident that the proposed additions to behavior therapy are appropriate for use with children with ADHD. However, the degree to which the identified differences in reinforcement sensitivity are specific to ADHD is largely unknown. The literature reviewed typically compares the performance of children with ADHD to TD controls, while not taking into account the potential influence of other common comorbid conditions in ADHD, e.g., ODD, anxiety disorders or no distinction is made between whether children display certain features (e.g., a comparison between children with ADHD who have heightened emotional lability and those who do not). Heightened emotional lability is often present among children with ADHD (Graziano and Garcia 2016), which may be exacerbated in the context of frustrating experiences such as punishment procedures in a behavioral parent training program. Although this needs to be tested empirically, this enhanced emotional state may hinder the ability to learn in the context of punishment procedures in a behavioral parent training program, especially for those children. Furthermore, there is very little research available on the reinforcement learning of groups presenting with different psychiatric disorders. Research comparing children with ADHD to children with ODD is especially relevant in this context, as BPT was developed for children with behavioral disorders in general. Thus, it is currently not clear how different comorbidities affect reinforcement learning in ADHD, some studies have explored the effect of reward on cognitive tasks, thereby comparing children with ADHD to children with ADHD and comorbid ODD (e.g., Tenenbaum et al. 2019; Van der Meere et al. 2005); however, research into effects of comorbidity on learning from reward and punishment is scarce. For the specific recommendation of limiting the use of punishment techniques in BPT for ADHD this may be especially relevant. In ODD samples lower punishment sensitivity is suggested (Matthys et al. 2004, 2012), with inclusion of time-out and natural consequences as punishment procedures in BPT being associated with enhanced effects of programs (Leijten et al. 2019). Our review suggests that for children with ADHD responses to punishment may be heightened thereby hindering them from further learning. Also similarly, to the best of our knowledge no empirical data are available on the impact of anxiety in ADHD on learning from reward and punishment. Children with anxiety disorders show higher temperamental sensitivity to punishment, making them more prone to detect and avoid punishing signals from the environment and enhanced feelings of anxiety in the context of punishment (Bijttebier et al. 2009). It has been suggested that the subgroup of children with ADHD and anxiety are characterized by high punishment sensitivity, which makes them potentially even less able to learn adaptive behavior in the context of punishment (Jarrett and Ollendick 2008; Nigg et al. 2004). Larger study samples allowing for subgroup analyses are needed to permit further refinement of behavior therapy for ADHD.

The current recommendations to adapt BPT and take account of altered reinforcement sensitivity in children with ADHD are based on our existing knowledge of these alterations. It is clear from reviewing the literature that altered reinforcement sensitivity in ADHD is subtle and complex. There is no doubt that in many ways the responses of children with ADHD to reward and punishment mirror those of TD children. Rewarding behavior increases the likelihood it will be repeated, mild punishment for inappropriate behavior reduces its frequency. As researchers continue to investigate the responsiveness of children with ADHD to reinforcement contingencies further adaptions to our recommended practices will undoubtedly be necessary.

## Footnotes

1 Not all elements included in our table were examined in this meta-analysis (e.g., response cost was not examined).

2 Gambling tasks are sometimes described in the literature as punishment tasks, however, as these tasks do not assess the effects of punishment alone, i.e., results are confounded by reward immediacy/size, and may reflect poor decision making (Furukawa et al. 2019a, b), we do not describe these here.
